# A Coarse-to-Fine Framework with Curvature Feature Learning for Robust Point Cloud Registration in Spinal Surgical Navigation

**DOI:** 10.3390/bioengineering12101096

**Published:** 2025-10-12

**Authors:** Lijing Zhang, Wei Wang, Tianbao Liu, Jiahui Guo, Bo Wu, Nan Zhang

**Affiliations:** 1 School of Biomedical Engineering, Capital Medical University, Beijing 100069, China; zhanglijing829@163.com (L.Z.); 122023010107@mail.ccmu.edu.cn (T.L.); guojiahui@mail.ccmu.edu.cn (J.G.); 2 Department of Orthopedics, Xuanwu Hospital Capital Medical University, Beijing 100053, China; wangwei1@xwhosp.org

**Keywords:** surgical navigation, pedicle screw fixation, cross-source point cloud, low overlapping ratio, point cloud registration

## Abstract

In surgical navigation-assisted pedicle screw fixation, cross-source pre- and intra-operative point clouds registration faces challenges like significant initial pose differences and low overlapping ratio. Classical algorithms based on feature descriptor have high computational complexity and are less robust to noise, leading to a decrease in accuracy and navigation performance. To address these problems, this paper proposes a coarse-to-fine registration framework. In the coarse registration stage, a Point Matching algorithm based on Curvature Feature Learning (CFL-PM) is proposed. Through CFL-PM and Farthest Point Sampling (FPS), the coarse registration of overlapping regions between the two point clouds is achieved. In the fine registration stage, the Iterative Closest Point (ICP) is used for further optimization. The proposed method effectively addresses the challenges of noise, initial pose and low overlapping ratio. In noise-free point cloud registration experiments, the average rotation and translation errors reached 0.34° and 0.27 mm. Under noisy conditions, the average rotation error of the coarse registration is 7.28°, and the average translation error is 9.08 mm. Experiments on pre- and intra-operative point cloud datasets demonstrate the proposed algorithm outperforms the compared algorithms in registration accuracy, speed, and robustness. Therefore, the proposed method can achieve the precise alignment of the surgical navigation-assisted pedicle screw fixation.

## 1. Introduction

Pedicle screw fixation is a common spinal surgical method for treating spinal fractures, lumbar spondylolisthesis and other diseases [1]. However, due to the spine’s complex structure and high precision requirements, the procedure is challenging for surgeons. Surgical navigation systems improve accuracy and safety by providing precise positioning, and are widely adopted in neuro-navigation [2,3,4], spinal [5,6], laparoscopic [7,8], knee replacement [9,10], and mandibular surgeries [11,12], playing an increasingly important role in various fields [13]. Registration is one of the core technologies in surgical navigation systems, and its precision is an important factor affecting the accuracy of the navigation system [14]. Therefore, many scholars are actively engaged in research to improve the accuracy of registration in navigation systems with a view to further optimizing the performance of the system [15,16,17]. In the study of registration applied to neuro-surgical navigation systems [15], surface registration based on automatic machine learning is proposed, which can improve the registration accuracy through experimental verification. In total knee arthroplasty assisted by a surgical navigation system [16], a point-to-surface iterative closest point registration algorithm is used, and the RMSE is 0.3 mm.

Surgical navigation-assisted pedicle screw fixation still faces challenges in achieving accurate the pre- and intra-operative registration. First, pre- and intra-operative point clouds from different imaging devices vary significantly in density and position. Second, intra-operative scanning limitations—such as limited anatomical exposure, soft tissue occlusion, and noise—reduce the low overlapping ratio between pre- and intra-operative point clouds. The point cloud registration algorithms in the above application will fail in this navigation-assisted context.

In terms of the existing classical feature extraction registration algorithms, Liu [18] proposed an algorithm integrating feature regions and Super4PCS for coarse registration under low overlap, which improves accuracy, success rate and reduces computation time compared to Super4PCS. Wang [19] developed a coarse registration algorithm using grid normal deviation angle statistics, improving alignment accuracy for partially overlapping point clouds. To address noise and low overlap, Zhang [20] proposed an SSR feature descriptor and design SSR-Net. The results demonstrated the effectiveness of the proposed method. Yan [21] proposed a hybrid approach combining SHOT with RANSAC for initial alignment, followed by symmetric ICP refinement, achieving clinically acceptable registration precision. Zhang [22] optimized registration by selecting FPS-based local regions for SAC-IA before ICP refinement, outperforming other methods in accuracy and success rate.

However, classic feature extraction-based point cloud registration algorithms have higher computational complexity, long execution times, and weaker noise robustness compared to deep learning algorithms [23,24,25,26,27]. To address this issue, Zhang [28] proposed an end-to-end network adapting focus on overlapping regions, and experiments proved it outperformed existing methods above 30% overlap and detects these regions accurately. Zhou [29] introduced SCANet, a spatial and channel attention network, which outperforms existing algorithms on partially overlapping point clouds with Gaussian noise. Li [30] proposed a transformer-based algorithm to address low overlap challenges. This algorithm employed a learnable geometric position update module and a deeper cross-attention module. Results demonstrate the proposed algorithm exhibits improvements over existing similar methods.

These methods do not consider both cross-source and a low overlapping ratio simultaneously, and cannot be directly applied to the pre- and intra-operative point clouds registration of pedicle screw fixation. Therefore, inspired by [31], this paper proposes a novel coarse-to-fine registration framework. The main contributions of this paper are as follows,

- A novel and robust coarse-to-fine registration framework has been proposed to address significant initial pose differences, low overlapping ratio, and noise interference issues.
- A novel Curvature Feature Learning-based Point Matching (CFL-PM) algorithm based on a curvature feature coder and graph attention network is proposed. The algorithm effectively generates more reliable correspondences for coarse registration and shows strong anti-interference ability against noise.
- A challenging dataset consisting of cross-source, low-overlapping pre- and intra-operative point cloud pairs to simulate real surgical environments. The noise-free conditions simulate an ideal surgical scenario, while noisy conditions simulate various noises present in the surgical field, such as soft tissues and blood. The results verified the feasibility and robustness of the proposed algorithm in the surgical navigation system.

The structure of this paper is as follows, Section 2 introduces the proposed algorithm. Section 3 describes the experimental setup, the results and the analysis. Section 4 concludes the paper.

## 2. Materials and Methods

**Figure 1:**
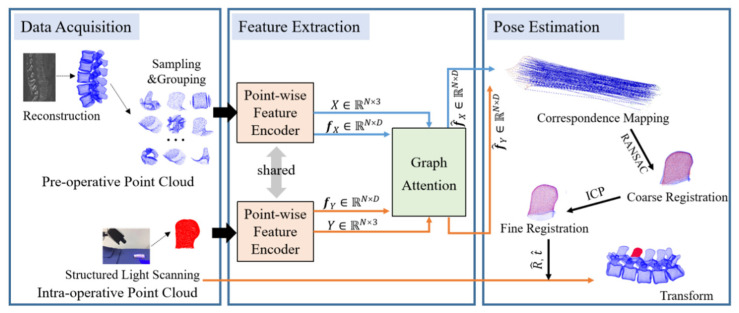
Pre- and intra-operative point clouds registration algorithm framework based on curvature feature encoder and graph attention network.

In surgical navigation system-assisted pedicle screw fixation, an important prerequisite for accurate intra-operative positioning is the alignment of the patient’s pre-operative point cloud with the actual intra-operative point cloud. The algorithmic framework proposed in this paper is shown in Figure 1.

The proposed algorithm identifies and registers the corresponding relationship by learning point features. During the training process, firstly, the intra-and the pre- with corresponding spines operative point clouds containing curvature information are input into the point-wise feature encoder, to obtain the key point subset X, Y and corresponding feature vectors $f_{X}$, $f_{Y}$.

Secondly, through the graph attention network, the updated point features ${\hat{f}}_{X}$, ${\hat{f}}_{Y}$ are obtained. And the generated features are used to determine the corresponding relationship between the points in the pre- and intra-operative point clouds. Thirdly, the Random Sample Consensus (RANSAC) method is used to delete the wrong matches from the matching relationship and perform coarse registration.

During testing, the pre-operative point cloud is sampled by FPS, and divided into multiple local regions. Based on the CFL-PM algorithm, registration errors between the intra-operative point cloud and each local region are compared to identify the highest-overlap region. The local region with the minimum error provides the coarse registration transformation. Finally, ICP is used for further optimization to obtain the optimal rotation matrix $\hat{R}$ and translation vector $\hat{t}$.

### 2.1. Curvature Feature Encoder

**Figure 2:**
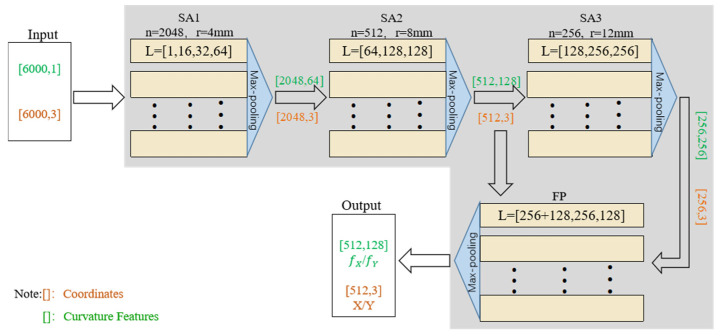
Point-wise feature encoder model architecture. (n: the number of output points; r: sampling radius; L: perceptron layers and nodes).

The curvature feature encoder consists of a point-wise feature encoder and a graph attention network [31]. The network structure of a point-wise feature encoder is shown in Figure 2, including three Set Abstraction (SA) layers SA1–SA3 and one Feature Propagation (FP) layer [32].

The input to the curvature feature encoder consists of 3D point coordinates and 1D corresponding features. The curvature value of the point is taken as the feature input, and its calculation is shown in Equation (1) [33,34],

(1) $$\sigma =\frac{\lambda_{3}}{\lambda_{1}+\lambda_{2}+\lambda_{3}}$$

where $\lambda_{1}$, $\lambda_{2}$, $\lambda_{3}$ are the eigenvalues of the covariance matrix of the point-fitting surface ($\lambda_{1}>\lambda_{2}>\lambda_{3}$). The curvature estimation neighborhood radius in this study is set to 5 mm.

The pre- and intra-operative point clouds are input to the encoder separately, and after the SA and FP layers, the coordinates of the sampled subsets (key-points) of the pre- and intra-operative point clouds are output, denoted by X and Y, respectively, as well as their respective feature vectors $f_{X}$, $f_{Y}$. Each SA layer does the FPS method for the points from the previous layer, which reduces the number of key points and improves the computational time without losing the information of the points on the overlapping region. The hyper-parameters *n*, *r*, and *L* in the SA layer are selected through several experiments to select the values corresponding to the time when the training effect is the best.

However, the output feature vectors $f_{X}$, $f_{Y}$ represent the local information, are not related to the global context. And the features of the points will change with the change in the neighborhood point density, which increases the difficulty of correctly identifying the correspondences. In order to reduce the impact of the above problems on registration, the graph attention network is added. Through the self-attention and cross-attention of the source and target point clouds [35], the updated point features ${\hat{f}}_{X}$, ${\hat{f}}_{Y}$ are obtained.

### 2.2. Correspondence Identification and Transformation Matrix Estimation

The correspondence is obtained by comparing the features of the key points X and Y of the source and target point clouds. Through the feature vectors ${\hat{f}}_{X}$, ${\hat{f}}_{Y}$, the similarity function (Equation (2)) of the key point matching represents the matching probability between X and Y,

(2) $$\phi =Softmax(\frac{{\hat{f}}_{X}\cdot {\hat{f}}_{Y}^{T}}{T})\in {\mathbb{R}}^{N\times N}$$

where *N* is the number of key points, *T* is a hyper-parameter, and the SoftMax function is a normalized exponential function. Each $\phi_{ij}$ represents the probability that the *i*-th source key point matches the *j*-th target key point, while each row $\phi_{i}$ represents its matching distribution across all target key points.

Next, considering that the two point clouds partially overlap, only some points have corresponding relationships. The noise of the scanner sensor and the difference in point density will also cause encoding errors and matching relationship errors. To reduce the influence of outliers, the RANSAC method is used and then estimates the transformation matrix.

The training process optimizes the encoder by minimizing the loss function [31] to maximize the match probability of the corresponding points and minimize the match probability of the non-corresponding points under the ground-truth transformation matrix to obtain the transformation matrix between the source and target point clouds and achieve coarse registration. The initial values of the hyper-parameters *T* in Equation (2) and in the loss function are set to 1 × 10^−2^ and 10, respectively.

### 2.3. Pre- and Intra-Operative Registration of Cross-Source and Low Overlapping Ratio Point Clouds

In practical applications, the low overlapping ratio and large density difference present a significant challenge to correctly identifying the correspondence and achieve successful alignment. To address this issue, the CFL-PM method is combined with the FPS to achieve pre- and intra-operative registration.

**Figure 3:**
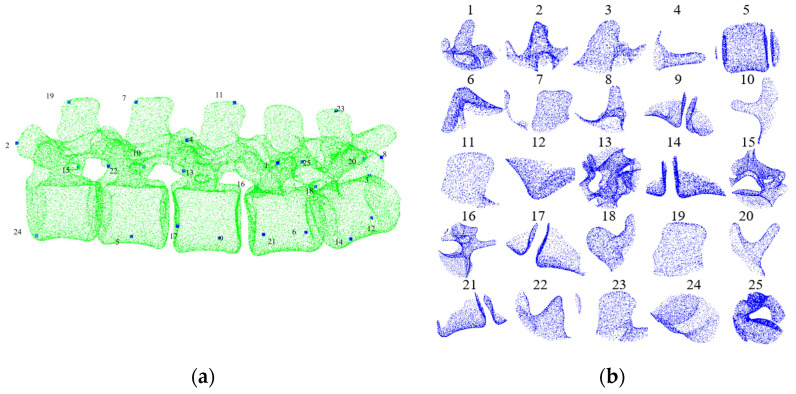
Pre-operative point cloud sampling points and the corresponding local regions. (**a**) Sampling points; (**b**) local area corresponding to sampling points.

Firstly, the intra-operative point cloud is down-sampled to achieve a density that is similar to the pre-operative point cloud. For the pre-operative point cloud *Q*, the FPS method is employed in order to obtain a set of sampling points *Q*′ (Figure 3a). A neighborhood search is then performed to divide the pre-operative point cloud into multiple local regions, with the local regions forming a candidate set (Figure 3b). The search radius is set to *r*, and for each point *p_i_* in the sampled points, its corresponding 3D region $P\left(p_{i}\right)$ can be expressed as Equation (3).

(3) $$P\left(p_{i}\right)=\left\{p|p\in Q,p_{i}\in Q^{\prime },p_{i}-p\leq r\right\}$$

Next, traverse the candidate set, based on the trained model, calculate the depth features of the local regions and the intra-operative point cloud, perform feature matching and pose estimation, and then calculate the rough alignment’s rotation error and translation error [36], as shown in Equations (4) and (5),

(4) $$\Delta T=T{\left(T^{G}\right)}^{-1}=\left[\begin{array}{cc} \Delta R & \Delta t\\ 0 & 1 \end{array}\right]$$

(5) $$\left\{\begin{array}{c} e^{r}=\arccos(\frac{tr\left(\Delta R\right)-1}{2})\\ e^{t}=\Delta t \end{array}\right.$$

Comparing the rotation error $e^{r}$ of the matching between the intra-operative point cloud and the local regions, as shown in Equation (6). The local region corresponding to the smallest error is the optimal local region, and its corresponding transformation is the transformation of the intra- and the pre-operative point clouds for coarse registration.

(6) $$\left\{E;T\right\}=\min\left\{e_{1}^{r},e_{2}^{r},\ldots ,e_{i}^{r},\ldots ,e_{N}^{r}\right\}\left(i=1,2,\ldots N\right)$$

The FPS and CFL-PM methods determine point correspondences to complete the coarse registration of the optimal local region with the intra-operative point cloud. Finally, ICP fine registration ensures better alignment despite large initial positional differences and low overlapping ratio, achieving cross-source, low-overlapping ratio pre- and intra-operative point cloud registration.

## 3. Results and Discussion

### 3.1. Evaluation Metrics

To assess the accuracy and robustness of the proposed algorithm for pre- and intra-operative point clouds registration, it is compared with three algorithms: ICP based on FPFH features (FPFH + ICP) [37], ICP based on SHOT features (SHOT + ICP) [38], and ICP based on FPS and FPFH (FPS + FPFH + ICP) [22]. The registration performance is gauged by rotation error $e^{r}$ and translation error $e^{r}$. Additionally, the runtime considered is the time taken for registering two point clouds, excluding the loading time for the point cloud data.

### 3.2. Model Training and Testing

**Figure 4:**
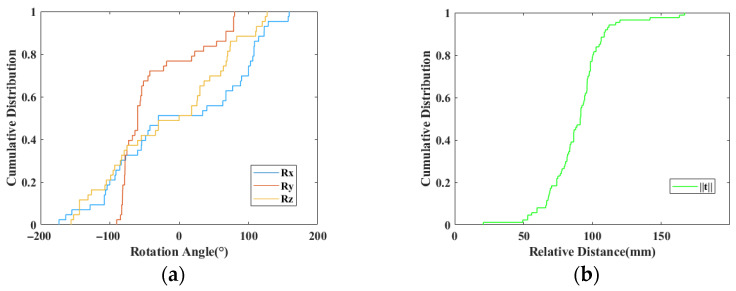
Cumulative distribution of rotation angles and relative distances between two point cloud coordinate systems. (**a**) Cumulative distribution of rotation angles; (**b**) cumulative distribution of relative distances.

The patients’ CT data used come from the SpineWeb Dataset [39,40] and the affiliated hospital, including lumbar vertebrae and cervical vertebrae. Pre-operative point cloud data is obtained through three-dimensional reconstruction of the patient’s pre-operative CT images. The reconstructed pre-operative CT are 3D printed. A COMET6 structured light scanner scanned the surgically exposed areas of the patient from the 3D printed phantom to simulate an actual intra-operative procedure to acquire intra-operative point cloud data. The intra-operative point cloud contained only the single spinous process. Figure 4 shows the cumulative distribution of point cloud pairs in the dataset in terms of rotation angle $R_{x}$, $R_{y}$, $R_{z}$, and translation distance $\left\|t\right\|$. The rotation angle and distance are obtained through ground-truth matrix. The ground-truth transformation matrix for the initial cross-source point cloud samples was established by performing a manual alignment. Then, the ground-truth transformation matrix was further refined by the ICP algorithm [41]. After data augmentation, we obtained 142 points cloud samples in total.

The dataset for training and validation comprised 142 samples. The dataset was divided into a training set and a validation set at a 3:1 ratio. Each sample consists of a pair of pre- and intra-operative point clouds and a ground-truth transformation matrix for registration. The dataset includes vertebrae with varying degrees of degeneration and anatomical morphology to ensure diversity. The test set comprises additional data, as shown in Table 1. Since the pre-operative point cloud is sampled at a frame rate of 25 local regions (as shown in Figure 3b), the intra-operative exposed spinous process point cloud must be sequentially matched with each segmented local region. Therefore, each test data point contains 25 sets of pre-intra operative point cloud pairs.

During the training process, the Adam optimizer is used, with the learning rate set to 1 × 10^−4^, and the learning rate is halved every five Epochs. The batch size and Epoch are set to 4 and 100, respectively.

**Figure 5:**
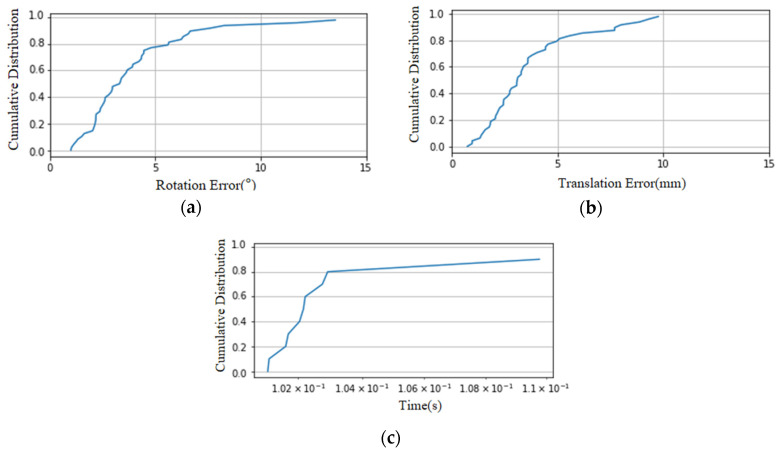
Cumulative distribution of rotation error, translation error, and coarse registration time on the validation set. (**a**) Cumulative distribution of rotation error; (**b**) cumulative distribution of translation error; (**c**) cumulative distribution of time.

To verify the trained model and the proposed algorithm, the results obtained are shown in Figure 5. It can be seen that the coarse registration achieves rotation error < 15°, translation error < 10 mm, and the registration time is approximately 0.1 s.

The operating system used in the experiment was Ubuntu 18.04, conducted on a NVIDIA Tesla V100 GPU with 32 GB memory and an Intel (R) Xeon (R) Silver 4116 CPU @ 2.10 GHz. The software environment includes CUDA 11.4, Python 3.9.12, and PyTorch 1.7.1.

### 3.3. Experiment on Pre- and Intra-Operative Registration of Cross-Source and Low Overlapping Ratio Point Clouds

In this experiment, the trained model registers intra- and pre-operative point clouds on a noise-free dataset, and evaluates the registration performance. The test data includes three cervical vertebrae and eight lumbar vertebrae cases. The pre-operative point clouds cover complete vertebrae, while intra-operative ones focus on surgically exposed areas. Table 1 details exposed sites, overlapping ratios, and initial pose differences between the pre- and intra-operative point clouds, providing insights into the registration effectiveness and challenges.

**Figure 6:**
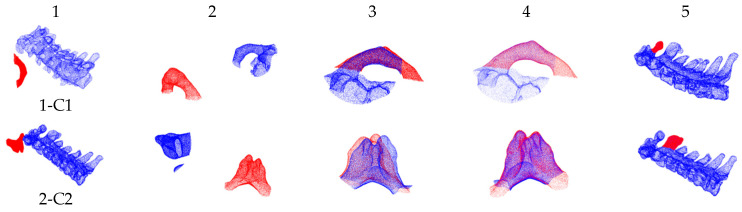

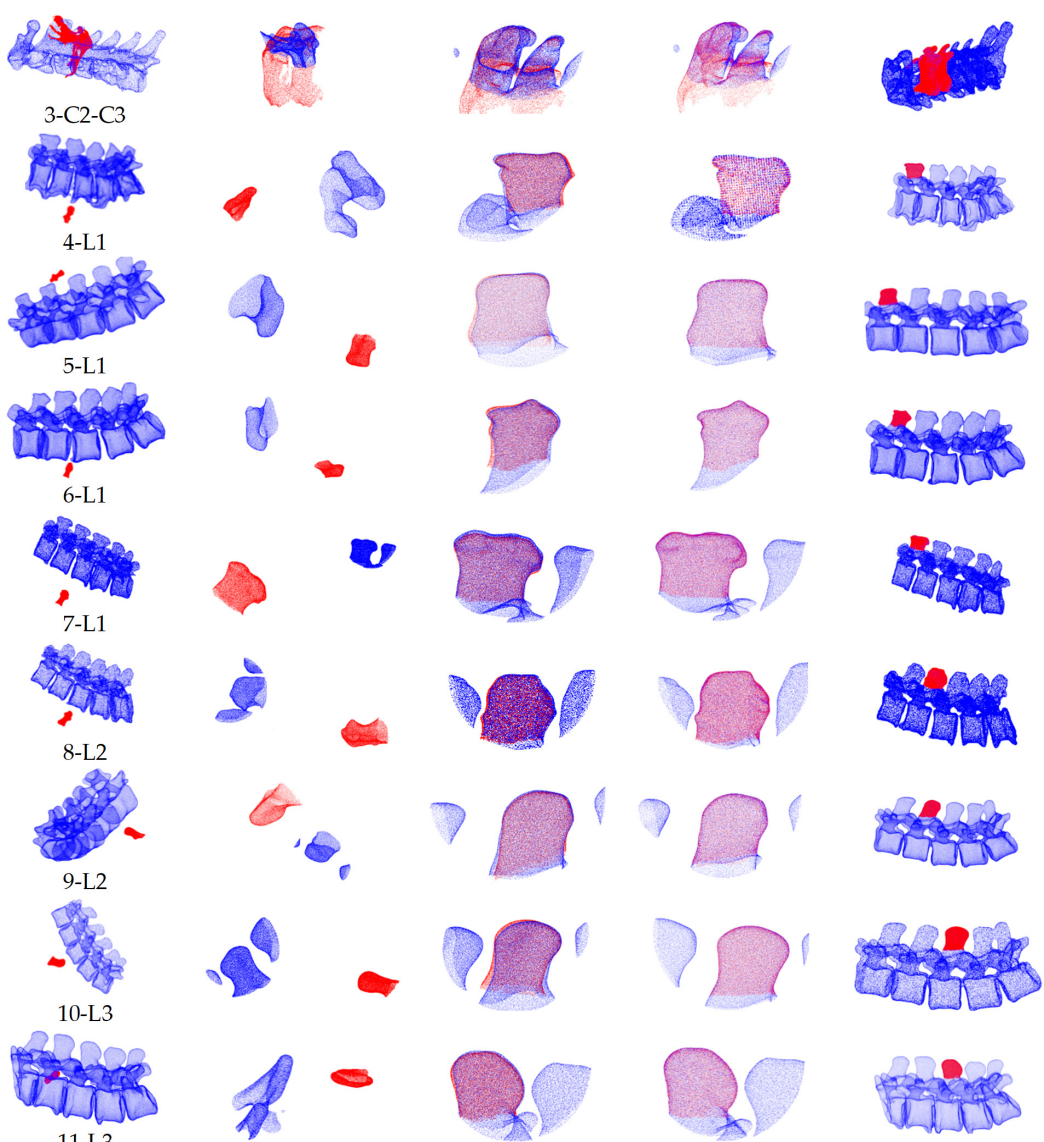
Registration results of pre- and intra-operative point clouds with various initial poses (red indicates intra-operative point cloud; blue indicates pre-operative point cloud).

Figure 6 illustrates the registration results of point cloud data from Table 1 under various initial poses. The first column displays the initial positions of the pre- and intra-operative point clouds. The second column shows the initial pose of the intra-operative point cloud and pre-operative spinous point cloud selected through FPS. The third column displays the coarse registration results obtained from the trained model. The fourth column reveals the fine registration results after ICP refinement. The fifth column visualizes the ultimate registration result.

Table 2 compares the accuracy and efficiency of the proposed algorithm and the other three algorithms. The results show that the proposed algorithm outperforms the others. Under the FPFH + ICP and SHOT + ICP, Data 1, 3, 6, and 8 cannot be successfully registered. Data 9 cannot be successfully registered under the FPFH + ICP. Data 11 and Data 4 fail to register under the SHOT + ICP algorithm. When the C1 spinous process is exposed in data 1, FPS + FPFH + ICP selected the best local area, but struggled with accurate alignment during the ICP stage. In contrast, the proposed algorithm successfully registered all datasets. For point cloud data that all can be successfully registered, at the coarse registration stage, the average rotation error and translation error of FPFH + ICP are 18.891° and 24.785 mm, respectively. With SHOT + ICP, the average rotation error is 20.392° and the translation error is 21.957 mm. The average rotation and translation errors of FPS + FPFH + ICP are 9.317° and 9.326 mm, respectively. Under the proposed algorithm, the average rotation error is 5.679°, and the translation error is 7.420 mm. The coarse registration error of the algorithm proposed is the smallest, which can provide a better initial pose for fine registration and accelerate convergence. The final average rotation error and translation error of the proposed algorithm are 0.342° and 0.268 mm, respectively, meeting clinical requirements. In terms of speed, proposed algorithm is the fastest, taking only 9.862 s on average, compared to much longer times for the other algorithms.

### 3.4. Robustness Evaluation

**Figure 7:**
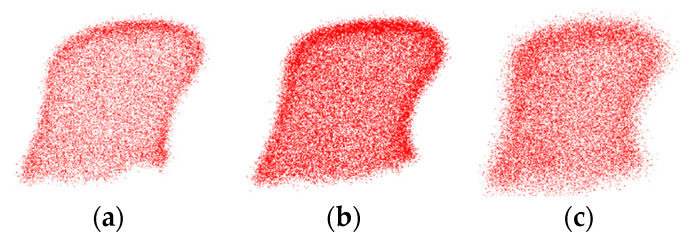
Intra-operative point clouds with different levels of Gaussian noise. (**a**) Point cloud with a standard deviation of 0.5 mm; (**b**) point cloud with a standard deviation of 0.75 mm; (**c**) point cloud with a standard deviation of 1.0 mm.

To evaluate the robustness of the proposed algorithm to noise, Gaussian noise with different standard deviations is added to the intra-operative point cloud, and compared with the registration algorithms based on FPFH and SHOT. The intra-operative point cloud used in the experiment still consists of scans from the exposed surgical site. The pre-operative point cloud data is manually extracted from the complete lumbar and cervical vertebrae point clouds, specifically targeting the spinous processes of the respective vertebrae. The initial positioning and orientation obtained from the previous experiment are maintained. The experimental data remains the same as the point cloud data presented in Table 1. Figure 7 visualizes the effects of adding noise with standard deviations of 0.5 mm, 0.75 mm, and 1.0 mm to intra-operative point cloud of dataset 1.

The experiment obtained the rotation error $e^{r}$ and translation error $e^{r}$ of pre- and intra-operative point clouds registration under the interference of different levels of standard deviation noise δ, as shown in Table 3. It can be seen from the results in the table that the registration errors $e^{r}$ of the three feature matching algorithms all increase with the increase in noise. Among them, both FPFH-based and SHOT-based coarse registration have results with errors greater than 60° or 60 mm. When the coarse registration error is less than 60, the mean coarse registration error under different δ is calculated as the smallest error of CFL-PM algorithm. The mean $e^{r}$ is 7.28°, and the mean $e^{r}$ of coarse registration is 9.08 mm. CFL-PM algorithm is stable for registration with or without noise, and has stronger robustness than the other two feature matching algorithms.

**Figure 8:**
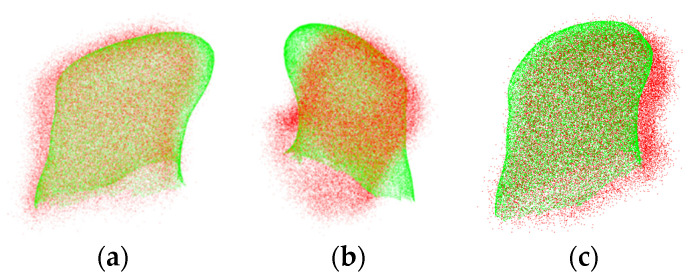
Coarse registration results with Gaussian noise standard deviation of 1. (**a**) Coarse registration based on SHOT; (**b**) coarse registration based on FPFH; (**c**) coarse registration based on CFL-PM. (Red indicates intra-operative point cloud after adding Gaussian noise; Green indicates pre-operative point cloud).

Figure 8 visualizes the pre- and intra-operation point clouds coarse registration results for Data 9 with Gaussian noise standard deviation of 1, which can be more intuitively seen that the proposed algorithm can improve the robustness of registration.

## 4. Conclusions

This study presented a novel coarse-to-fine registration framework to address the critical challenges of large initial pose differences, low overlap, and noise in surgeon-navigated pedicle screw placement. The proposed CFL-PM algorithm, rooted in curvature feature learning, demonstrated its efficacy as a robust solution for the critical coarse registration stage. The experimental results confirmed that the proposed method achieves superior performance in terms of accuracy (e.g., 0.34° rotation error and 0.27 mm translation error under ideal conditions), computational efficiency, and robustness to noise. Besides the technical metrics of accuracy and speed, the proposed registration framework holds potential impact on enhancing clinical workflows in spinal surgery. For patient counseling, surgeons can more effectively explain the surgical plans, thereby helping to alleviate patient anxiety. For intra-operative surgical judgment, the proposed method showed the ability to achieve robust and precise registration between pre- and intra-operative point clouds, even under challenging conditions of noise and low overlap. The surgeons can more confidently make decisions based on the pre-determine the optimal pedicle screw trajectory. Daly [42] proposed a markerless tracking system based on a clinical RGB-D camera, which is capable of capturing the point cloud of the intra-operative spine. The proposed CFL-PM algorithm can be utilized in this scenario to extract patient-specific spinal features, to achieve precise registration between pre-operative and intra-operative point clouds. This enables the augmented visualization of the patient’s surgical field during surgery with surgical navigation-assisted pedicle screw fixation, allowing the procedure to be executed according to the screw trajectory planned pre-operatively.

Despite these encouraging results, several limitations of the current work should be acknowledged. The dataset used although is sufficient for the algorithm, further validation through prospective clinical trials and larger datasets is necessary to establish its generalization in different patient groups and surgical environments. Future work may focus on integrating this algorithm into a clinical navigation platform and validating its benefits through clinical studies. At the same time, its applicability in other orthopedic navigation procedures beyond the placement of pedicle screws will also be explored.

## Figures and Tables

**Table 1:** Exposed sites and initial pose differences between pre- and intra-operative point clouds.

Data	Exposed Site	Overlapping Ratio (%)	Rotation Angles (°)			Relative Distances (mm)		
			X	Y	Z	X	Y	Z
1	C1	2.04	−93.94	−83.85	150.77	3.63	21.99	−31.57
2	C2	1.98	78.07	−51.48	10.12	−48.17	60.95	37.35
3	C2–C3	4.27	−169.52	−55.43	−136.57	5.81	6.19	33.71
4	L1	1.35	−76.59	68.13	−88.32	−98.28	−59.36	26.07
5	L1	1.66	89.39	58.15	82.35	−57.78	−42.73	38.71
6	L1	1.67	96.94	22.41	82.55	−89.11	45.34	42.65
7	L1	1.79	80.76	−62.50	−48.06	−52.53	110.76	−86.79
8	L2	1.53	89.98	−73.34	−78.47	6.99	102.23	0.95
9	L2	1.69	148.53	48.47	138.47	114.08	−64.99	−103.52
10	L3	1.85	161.82	−83.28	−116.87	−4.17	−83.28	−91.19
11	L3	2.29	109.48	28.19	79.30	−55.67	7.23	40.84

**Table 2:** Comparison of registration errors and runtime for pre- and intra-operative point clouds under different algorithms.

Data	Exposed Site	Algorithm	Coarse Registration			Fine Registration		
			$e^{r}$ (°)	$e^{t}$ (mm)	Time (s)	$e^{r}$ (°)	$e^{t}$ (mm)	Time (s)
1	C1	[37] + ICP	/	/	/	/	/	/
		[38] + ICP	/	/	/	/	/	/
		[22]	23.75	29.42	102.95	/	/	/
		Proposed	10.11	19.04	8.11	0.43	0.37	0.33
2	C2	[37] + ICP	36.64	44.52	27.56	0.43	0.23	0.40
		[38] + ICP	29.37	15.03	37.56	0.32	0.31	0.34
		[22]	7.43	8.97	115.30	0.21	0.37	0.20
		Proposed	6.32	7.28	7.89	0.30	0.24	0.25
3	C2–C3	[37] + ICP	/	/	/	/	/	/
		[38] + ICP	/	/	/	/	/	/
		[22]	18.89	23.73	124.56	1.07	0.83	0.47
		Proposed	2.30	0.70	9.85	0.43	0.36	0.30
4	L1	[37] + ICP	19.82	40.49	47.01	0.99	0.41	0.35
		[38] + ICP	20.62	35.22	64.90	/	/	/
		[22]	8.43	4.84	115.18	0.25	0.85	0.08
		Proposed	4.03	3.91	10.18	0.38	66	0.09
5	L1	[37] + ICP	2.71	1.16	43.25	0.28	0.27	0.29
		[38] + ICP	3.02	1.81	57.61	0.41	0.58	0.31
		[22]	2.79	3.00	108.35	0.17	0.19	0.05
		Proposed	5.36	7.38	10.68	0.37	0.10	0.04
6	L1	[37] + ICP	/	/	/	/	/	/
		[38] + ICP	/	/	/	/	/	/
		[22]	4.12	8.51	113.54	0.59	0.60	0.06
		Proposed	4.52	5.54	9.84	0.38	0.41	0.04
7	L1	[37] + ICP	10.28	26.16	26.96	0.33	3.50	0.45
		[38] + ICP	12.84	14.71	46.39	0.39	1.52	0.57
		[22]	1.60	1.26	129.48	0.88	0.51	0.15
		Proposed	6.83	11.45	9.88	0.30	0.25	0.18
8	L2	[37] + ICP	/	/	/	/	/	/
		[38] + ICP	/	/	/	/	/	/
		[22]	4.84	3.11	115.44	0.31	0.47	0.31
		Proposed	5.93	5.86	9.96	0.49	0.11	0.21
9	L2	[37] + ICP	/	/	/	/	/	/
		[38] + ICP	36.45	43.43	58.97	0.60	0.78	0.69
		[22]	14.81	9.81	92.30	0.45	0.32	0.07
		Proposed	6.89	7.99	11.10	0.31	0.16	0.06
10	L3	[37] + ICP	35.13	26.52	47.65	0.33	0.38	0.44
		[38] + ICP	19.97	21.54	55.70	0.71	0.66	0.40
		[22]	9.27	9.80	117.78	0.35	0.32	0.26
		Proposed	2.76	4.21	9.75	0.26	0.14	0.26
11	L3	[37] + ICP	8.78	9.86	44.42	1.00	0.41	0.35
		[38] + ICP	/	/	/	/	/	/
		[22]	8.21	3.54	89.544	0.25	0.85	0.08
		Proposed	7.43	8.94	9.486	0.13	0.13	0.07

Note: “/” indicates registration failure under the current algorithm.

**Table 3:** Comparison of coarse registration errors with different standard deviation noise.

Data	Exposed Site	Algorithm	*δ* = 0.25 mm		*δ* = 0.5 mm		*δ* = 0.75 mm		*δ* = 1.0 mm	
			$e^{r}$ (°)	$e^{t}$ (mm)	$e^{r}$ (°)	$e^{t}$ (mm)	$e^{r}$ (°)	$e^{t}$ (mm)	$e^{r}$ (°)	$e^{t}$
1	C1	[37]	27.32	22.97	28.89	23.18	/	/	/	/
		[38]	35.42	25.16	27.83	14.04	52.35	20.30	40.86	21.12
		CFL-PM	4.21	7.81	8.76	15.70	12.28	20.57	17.26	20.20
2	C2	[37]	32.64	17.49	33.99	14.58	/	/	/	/
		[38]	36.85	20.71	/	/	/	/	/	/
		CFL-PM	6.36	5.99	16.32	5.85	20.18	10.03	25.24	8.51
3	C2–C3	[37]	11.25	3.81	20.66	6.28	/	/	/	/
		[38]	23.61	5.45	23.38	8.43	18.48	6.20	23.01	9.20
		CFL-PM	7.61	5.03	11.68	5.96	7.52	1.72	20.49	9.01
4	L1	[37]	33.32	12.02	20.08	25.96	41.76	23.17	31.08	46.65
		[38]	/	/	/	/	/	/	/	/
		CFL-PM	3.22	3.08	2.32	2.20	3.93	4.07	7.55	10.19
5	L1	[37]	/	/	/	/	/	/	/	/
		[38]	/	/	/	/	/	/	/	/
		CFL-PM	4.73	6.61	2.24	2.58	3.52	1.64	9.58	16.90
6	L1	[37]	5.78	1.44	15.12	4.23	36.62	7.30	/	/
		[38]	3.97	0.98	52.64	6.93	15.50	1.73	21.63	7.08
		CFL-PM	3.24	0.30	3.99	1.21	3.10	5.04	6.26	2.84
7	L1	[37]	20.28	24.90	46.56	27.81	19.68	24.23	/	/
		[38]	13.91	16.95	35.54	24.44	6.40	14.67	/	/
		CFL-PM	8.48	14.73	2.49	11.77	5.88	11.11	7.60	24.5
8	L2	[37]	10.77	4.11	21.12	14.54	21.09	14.35	/	/
		[38]	7.42	6.34	12.54	5.52	9.02	7.93	20.51	15.04
		CFL-PM	2.52	2.10	4.56	3.46	5.54	6.99	5.66	9.31
9	L2	[37]	26.79	13.78	22.16	16.58	33.39	15.64	21.18	16.43
		[38]	14.41	5.40	13.98	6.80	35.32	19.01	23.75	14.79
		CFL-PM	1.79	1.40	3.57	1.06	5.18	9.19	6.28	9.09
10	L3	[37]	48.27	47.20	47.43	43.02	19.8	33.28	52.49	46.78
		[38]	22.96	31.99	15.56	36.17	55.52	54.19	34.00	44.69
		CFL-PM	8.98	28.11	2.37	24.78	6.63	31.85	5.26	29.11
11	L3	[37]	10.78	7.30	16.41	2.27	26.03	7.52	52.79	10.39
		[38]	9.67	5.71	5.49	0.77	6.45	1.20	16.98	3.18
		CFL-PM	4.27	0.48	9.43	1.45	3.41	1.57	8.98	4.67
Mean coarse registration error		[37]	22.72	15.50	27.24	17.85	28.34	17.93	39.38	30.06
		[38]	18.69	13.19	23.37	12.89	24.88	15.65	25.82	16.44
		CFL-PM	5.04	6.88	6.16	6.91	7.02	9.43	10.92	13.12

Note: When the rotation error is greater than 60° or the translation error is greater than 60 mm, use “/” instead.

## Data Availability

The data are available from the corresponding author upon reasonable request, subject to the approval of the Medical Ethics Committee of Capital Medical University.
